# Regulatory Divergence as a Mechanism for X-Autosome Incompatibilities in *Caenorhabditis* Nematodes

**DOI:** 10.1093/gbe/evad055

**Published:** 2023-04-04

**Authors:** Athmaja Viswanath, Asher D Cutter

**Affiliations:** Department of Ecology and Evolutionary Biology, University of Toronto, ON, Canada; Department of Ecology and Evolutionary Biology, University of Toronto, ON, Canada

**Keywords:** gene regulation, speciation, *cis*-regulatory elements, hybrid male sterility, Dobzhansky–Muller incompatibilities, Haldane’s rule, hybrid transcriptome

## Abstract

The world's astounding biodiversity results from speciation, the process of formation of distinct species. Hybrids between species often have reduced fitness due to negative epistatic interactions between divergent genetic factors, as each lineage accumulated substitutions independently in their evolutionary history. Such negative genetic interactions can manifest as gene misexpression due to divergence in gene regulatory controls from mutations in *cis*-regulatory elements and *trans*-acting factors. Gene misexpression due to differences in regulatory controls can ultimately contribute to incompatibility within hybrids through developmental defects such as sterility and inviability. We sought to quantify the contributions of regulatory divergence to postzygotic reproductive isolation using sterile interspecies hybrids of two *Caenorhabditis* nematodes: *Caenorhabditis briggsae* and *Caenorhabditis nigoni*. We analyzed previous transcriptome profiles for two introgression lines with distinct homozygous X-linked fragments from *C. briggsae* in a *C. nigoni* genomic background that confers male sterility, owing to defects in spermatogenesis (Li R, et al. 2016. Specific down-regulation of spermatogenesis genes targeted by 22G RNAs in hybrid sterile males associated with an X-chromosome introgression. Genome Res. 26:1219–1232). Our analysis identified hundreds of genes that show distinct classes of nonadditive expression inheritance and regulatory divergence. We find that these nonoverlapping introgressions affect many of the same genes in the same way and demonstrate that the preponderance of transgressive gene expression is due to regulatory divergence involving compensatory and joint effects of *cis-* and *trans*-acting factors. The similar transcriptomic responses to nonoverlapping genetic perturbations of the X-chromosome implicate multiway incompatibilities as an important feature contributing to hybrid male sterility in this system.

**Significance**

The genetic causes of intrinsic postzygotic reproductive isolation can arise from hybrids experiencing negative gene regulatory interactions. In *Caenorhabditis* nematodes, hybrid male sterility involves X-autosome incompatibilities that affect small-RNA regulatory pathways. We sought to understand the role of gene regulatory divergence as a related contributor to hybrid misexpression by analyzing transcriptomes of sterile males from two hybrid introgression lines, each containing distinct X-linked fragments from *Caenorhabditis briggsae* in a *Caenorhabditis nigoni* genomic background. We show that gene misexpression occurs due to extensive joint divergence of *cis-* and *trans*-acting regulatory factors and provide evidence for multiway incompatibilities as an important feature of this system.

## Introduction

Speciation is the process of formation and maintenance of distinct species through the evolution of reproductive isolation. Events that split populations into separated groups, with restricted gene flow between those groups, can eventually lead to evolution of genetically intrinsic reproductive isolation capable of maintaining these groups as distinct species (Coyne and Orr 2004). Intrinsic postzygotic barriers take the form of developmental defects like hybrid sterility or hybrid inviability that can result from dysfunctional genotype–genotype interactions (Coyne and Orr 1998; Wolf et al. 2010; Coughlan and Matute 2020; Cutter and Bundus 2020). Multiple genetic mechanisms can create such incompatibility, including chromosomal rearrangements, gene loss and duplication, repetitive transposable element composition and activity, sequence pairing problems due to divergence, and negative epistatic interactions between two or more loci (i.e., Dobzhansky–Muller incompatibilities [DMIs]) (Orr 1995; Presgraves 2010; Maheshwari and Barbash 2011). Such hybrid incompatibilities can involve distinct genomic compartments, including cytonuclear incompatibilities or X-autosome incompatibilities (Turelli and Moyle 2007; Bundus et al. 2018). DMIs also may be genetically simple or require more complex interactions of three or more genetic factors, with such multiway incompatibilities expected to accumulate more rapidly over time (Orr 1995; Kondrashov 2003; Satokangas et al. 2020). Incompatibilities also can arise from protein–protein interactions or from interactions involving gene regulatory regions that yield misexpression in hybrids (Mack and Nachman 2017). The importance of regulatory divergence as an underlying mechanism of hybrid incompatibility, however, remains to be fully elucidated, especially with respect to sex biases in hybrid dysfunction (Mack and Nachman 2017).

The heterogametic sex, males in *Caenorhabditis* nematodes and many other taxa, is disproportionately afflicted by F1 hybrid sterility and inviability (Delph and Demuth 2016; Cutter 2018). Despite the prevalence of this phenomenon, termed Haldane's rule (Haldane 1922), there is limited consensus for a leading cause among the several hypotheses proposed to explain it (including dominance theory, faster X theory, faster male theory, and meiotic drive) (Wolf et al. 2010; Delph and Demuth 2016). To the extent that the developmental origins of Haldane's rule involve gene misexpression, regulatory divergence may play a crucial role in the manifestation of sex-biased hybrid incompatibility (Mack and Nachman 2017). Moreover, a wide range of taxa shows significant misexpression in interspecies hybrids of male-biased genes (Michalak 2003; Ranz et al. 2004) and spermatogenesis-related genes (Sundararajan and Civetta 2011; Ferguson et al. 2013), and disproportionate misexpression of X-linked genes in sterile hybrids (Lu et al. 2010; Oka and Shiroishi 2014; Gomes and Civetta 2015). These patterns of gene expression suggest that regulatory divergence of sex-biased genes and X-linked genes is instrumental in producing the dysfunction of developmental programs that lead to hybrid sterility (Coyne and Orr 2004; Llopart 2012; Coolon et al. 2015).

Misexpression of a given gene in hybrids, which we define as transgressive expression in a hybrid that exceeds the range of either parent, can arise due to divergence in regulatory controls caused by mutations in *cis*-regulatory elements encoded close to the gene and by changes in *trans*-acting factors that are encoded elsewhere in the genome (Landry et al. 2007; Mack and Nachman 2017). In F1 hybrids, in which all alleles experience a common *trans*-acting environment, any difference in expression between the two alternate alleles of a gene (allele-specific expression) indicates that *cis*-regulatory differences exist between the parents at that locus. If, instead, the gene's expression differs in F1 hybrids compared with the parents in the absence of F1 allele-specific expression differences, then, divergence of *trans*-acting factors must be the cause (Wittkopp et al. 2004). Commonly, however, studies of hybrids find that *cis*-acting changes are compensated by changes in *trans*-acting factors despite similar overall gene expression between species (Takahasi et al. 2011; Goncalves et al. 2012; Mack and Nachman 2017). Compensatory *cis-trans* regulatory evolution like this represents one way that molecular evolution can accrue despite stabilizing selection generally being pervasive that yields conserved overall gene expression levels between species (Gilad et al. 2006; Signor and Nuzhdin 2018). This approach of assessing overall and allele-specific expression with hybrid organisms has permitted quantification of genome-wide *cis-* and *trans-*acting regulatory divergence in plants, fungi, insects, mice, and nematodes, demonstrating important contributions of both *cis-*only and *trans*-only changes as well as both compensating and reinforcing joint effects of *cis*- and *trans*-acting changes (Wittkopp et al. 2004; Landry et al. 2005; McManus et al. 2010; Goncalves et al. 2012; Bell et al. 2013; Barrière and Ruvinsky 2014; Graze et al. 2014; Shen et al. 2014; Oka and Shiroishi 2014; Li and Fay 2017; Sánchez-Ramírez et al. 2021). Simulations predict that hybrid incompatibilities evolve rapidly under selection (Mack and Nachman 2017). Specifically, stabilizing selection leads to a slower accumulation of incompatibilities when arising from compensating regulatory changes, while directional selection results in a faster accumulation of incompatibilities (Johnson and Porter 2000; Tulchinsky et al. 2014). Stabilizing selection maintains a mean, nonextreme gene expression phenotype within a species, and during regulatory divergence, compensating *cis*-*trans* interactions can help reestablish overall gene expression levels (Signor and Nuzhdin 2018). Although there is widespread evidence that stabilizing selection is an important force underlying the evolution of gene expression, the relative contribution of divergence in *cis*-*trans* elements and compensating *cis*-*trans* interactions remains to be fully elucidated (Lemos et al. 2005; Gilad et al. 2006; Bedford and Hartl 2009; Signor and Nuzhdin 2018).

We sought to expand the logic of F1 hybrid inference of *cis*- and *trans*-acting divergence to investigate regulatory divergence in homozygous hybrid introgression lines of the nematodes *Caenorhabditis briggsae* and *Caenorhabditis nigoni* that show male sperm fertility defects. *C. briggsae* and *C. nigoni* diverged ∼3.5 Ma (35 × 10^6^ generations) (Cutter and Choi. 2010; Thomas et al. 2015) and represent the first sister species in this group discovered to produce viable and fertile F1 hybrids (Woodruff et al. 2010; Baird and Seibert 2013; Felix et al. 2014). These sister species have different reproductive modes: *C. briggsae* includes self-fertile XX hermaphrodites and XO males whereas *C. nigoni* is gonochoristic (XX females and XO males) (Haag 2005; Gupta et al. 2007). The F1 hybrids exhibit both Haldane's rule and Darwin's corollary to Haldane's rule with hybrid males always sterile and/or nonviable and viable hybrids produced only when *C. briggsae* is the maternal parent (Woodruff et al. 2010; Kozlowska et al. 2012 ; Bundus et al. 2015). Previous work on identifying incompatible loci in these hybrids found that some *C. nigoni* strains carrying introgression from *C. briggsae* have reduced fitness, although *C. nigoni* is generally more robust to introgression from *C. briggsae* than vice versa (Bi et al. 2015, 2019; Li et al. 2016; Xie et al. 2022). Genomic changes associated with the transition in reproductive mode, and divergence more generally, can potentially influence gene regulatory mechanisms. Specifically, X-linked introgressions from *C. briggsae* into a *C. nigoni* genomic background represent genetic perturbations that may disrupt the transcriptome in ways that permit the inference of *trans*-only and joint *cis-trans* regulatory divergence affecting genes in distinct ways. With this goal, we reanalyzed the transcriptome data set of Li et al. (2016) for males of two X-linked hybrid introgression lines for which ∼95% of the genome derives from *C. nigoni*, including all autosomes. Spermatogenesis-related genes are downregulated on the autosomes of these lines, and 22G RNAs targeted to spermatogenesis genes are upregulated, supporting an incompatible interaction between the X-chromosome and autosomes that involves perturbation of small RNA-mediated regulation (Li et al. 2016). Here, we augment these observations by inferring the gene regulatory divergence involving *cis*- and *trans*-acting changes on a per-locus basis that underlie X-autosome interactions and contribute to gene misexpression.

## Results

### Independent X-Linked Introgressions Disrupt the Transcriptome in Similar Ways

**Fig. 1.**
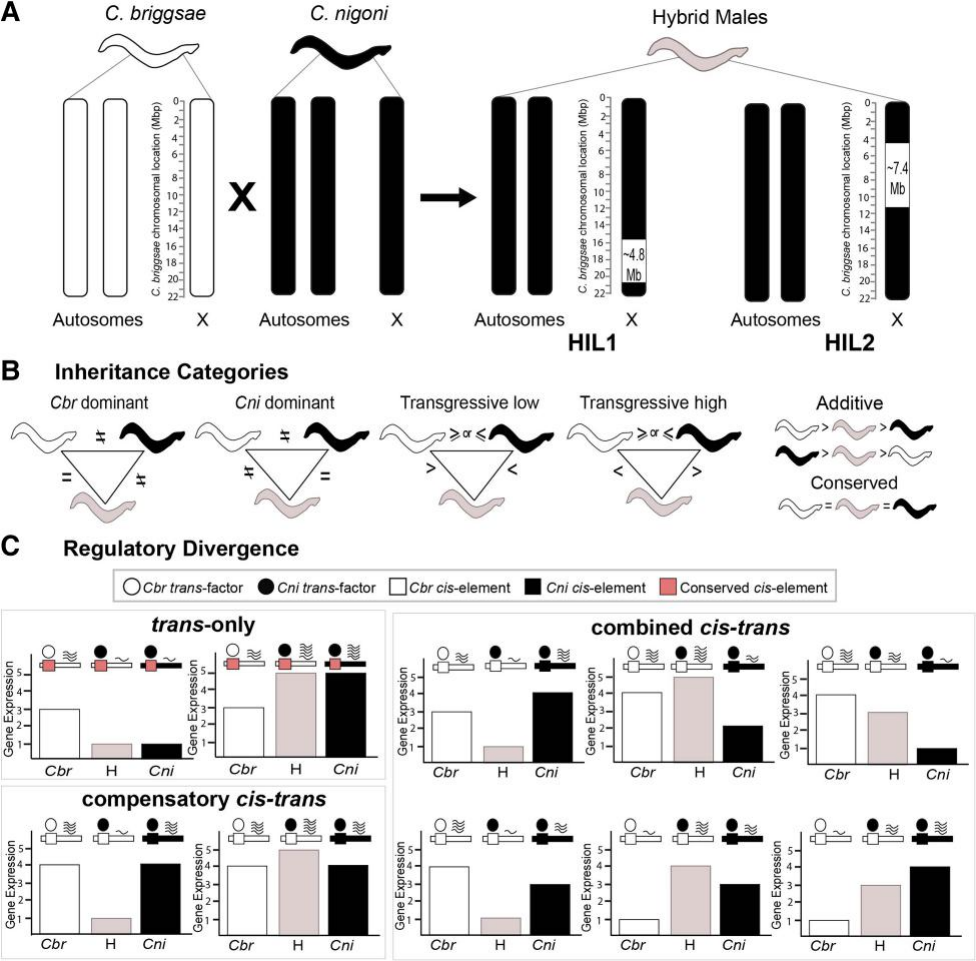
Classification of inheritance and regulatory divergence categories for genes in the shared genomic region. (*A*) HIL1 and HIL2 have ∼4.8 Mb and ∼7.4 Mb regions, respectively, on the X-chromosome introgressed from *C. briggsae* (white) in *C. nigoni* background (black). (*B*) Using differential gene expression analysis, genes were classified into five different inheritance categories based on a three-way comparison of expression between *C. briggsae*, *C. nigoni*, and hybrids: 1) Genes showing expression equal to one of the wild-type species that also showed significant difference between the two parent species were termed *C. briggsae* or *C. nigoni* “dominant”, 2) and 3) genes showing expression significantly different and below or above both wild-type species were termed “transgressive low” and “transgressive high,” respectively, 4) genes showing expression significantly different between the parents and significantly different but intermediate between both parents in HIL were termed “additive,” and 5) genes showing no significant differences in expression relative to wild-type gene expression were termed conserved. All other expression patterns were termed “ambiguous.” Equal signs indicate nonsignificant expression differences. (*C*) Pairwise contrasts of differential gene expression analysis between *C. briggsae*, *C. nigoni*, and HILs were used to infer three distinct regulatory divergence profiles. 1) *C. nigoni* expression differing significantly from *C. briggsae* and the HIL, in the absence of significant expression difference between HIL and *C. briggsae*, was classified as *trans-*only regulatory divergence. This is because when genes in *C. nigoni*, *C. briggsae*, and the HIL differ only in the *trans*-acting factor and have a common *cis*-element (pink square), the underlying *cis–trans* interaction in HILs is similar to interaction within *C. briggsae*. 2) When the gene in the HIL showed significant differential expression with both *C. nigoni* and *C. briggsae* genes, despite *C. nigoni* and *C. briggsae* displaying no significant difference between one another, it was classified as compensatory *cis–trans* regulatory divergence. 3) When all three pairwise contrasts displayed significant expression differences from one another, it was classified as combined *cis-* and *trans-*regulatory divergence. These combined effects could be compensatory, reinforcing, or a mixture of both. When genes in a HIL showed no significant expression difference with *C. nigoni* but showed significant differential expression with *C. briggsae* in the presence of significant differential gene expression between *C. nigoni* and *C. briggsae*, they were classified as having no effect. When the genes showed no significant differential expression in any pairwise contrast, they were classified as conserved, and all other expression circumstances were classified as ambiguous regulatory divergence. Different *cis-*elements (squares) and *trans*-acting factors (circles) may be encoded by *C. briggsae* (white) and *C. nigoni* (black). The conserved *cis*-element is represented by a pink square that is common to both *C. briggsae* and *C. nigoni*.

**Fig. 2.**
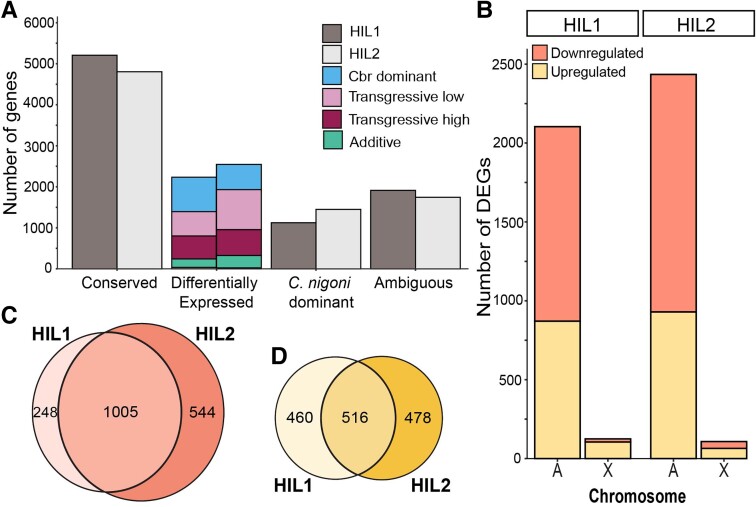
Shared patterns of differential expression by hybrid introgression lines. (*A*) Distribution of genes among differential expression designations for genes encoded within the portion of the genome shared by the HILs (i.e., excluding genes in the X-linked introgression regions). HIL2 (strain ZZY10307) included 68 more genes than HIL1 (strain ZZY10330) with detectable expression in this shared portion of the genome (*n*_HIL1_ = 10,473; *n*_HIL2_ = 10,541). Most genes showed a conserved expression profile in both HILs (*n*_HIL1_ = 5,207 of 10,473; *n*_HIL2_ = 4,803 of 10,541), with another 11% and 14% of genes with expression equivalent to *C. nigoni* wild-type expression in HIL1 and HIL2, respectively (*n*_HIL1_ = 1,124 of 10,473; *n*_HIL2_ = 1,450 of 10,541). Differentially expressed genes constituted 21% and 24% of total genes expressed in the common genome for HIL1 and HIL2, respectively (*n*_HIL1_ = 2,229 of 10,473; *n*_HIL2_ = 2,543 of 10,541). However, 18% and 17% of the genes in HIL1 and HIL2, respectively, did not fall into any of these categories and were classified as ambiguous (*n*_HIL1_ = 1,913 of 10,473; *n*_HIL2_ = 1,745 of 10,541). (*B*) Downregulated differentially expressed genes (orange) are more abundant than upregulated genes (yellow) in the common genomic region across the autosomes in both HILs, with few differentially expressed genes linked to nonintrogressed regions of the X-chromosome (*n*_HIL1_ = 1,253 downregulated genes of 2,229 DEGs among 10,473 total genes; *n*_HIL2_ = 1,549 downregulated genes of 2,543 DEGs among 10,541 genes). Upregulated and downregulated differential gene expression is defined based on expression in HILs relative to *C. nigoni*. Downregulated genes are enriched on autosomes relative to the X-chromosome in both HILs (Fisher's exact test *P*_HIL1_ < 2.2 × 10^−16^; *P*_HIL2_ = 7.1 × 10^−6^). (*C*) Of the 1,253 and 1,549 downregulated genes present on the shared genomic region in HIL1 and HIL2, respectively, a majority of genes overlapped in identity between HIL1 and HIL2 (1,005 of 1,253 downregulated genes of HIL1 or 1,005 of 1,549 downregulated genes of HIL 2; Fisher's exact test *P*_overlap_ < 0.001; Jaccard index = 0.6). (*D*) Of the 976 and 994 genes upregulated in HIL1 and HIL2, respectively, a majority overlapped in identity between HIL1 and HIL2 (516 of 976 upregulated genes in HIL1 or 516 of 994 upregulated genes in HIL2; Fisher's exact test *P*_overlap_ < 0.001; Jaccard index = 0.4).

To investigate the impacts that nonoverlapping introgressions of X-linked DNA from one species (*C. briggsae*) into another (*C. nigoni*) exert on the male transcriptome of genes encoded elsewhere across the genome (fig. 1), we quantified differential gene expression from the data reported by Li et al. (2016). We mapped reads using reference genomes of both *C. nigoni* and *C. briggsae* to test for differential expression and misexpression in hybrid introgression lines (HILs) relative to the parental species (cf. the analysis of Li et al. (2016), for which only the *C. briggsae* reference genome was available). We observed that most genes are expressed at similar levels across *C. nigoni*, *C. briggsae*, and hybrid introgression lines for the shared portion of the genome, that is, *C. nigoni* genes encoded outside of the X-linked introgression regions, consistent with prior reports (Li et al. 2016) (*n*_HIL1_ = 5,207 of 10,473 genes; *n*_HIL2_ = 4,803 of 10,541 genes). However, the HILs showed differential expression for 21% and 24% of genes in HIL1 and HIL2, respectively (*n*_HIL1_ = 2,229 of 10,473; *n*_HIL2_ = 2,543 of 10,541) (fig. 2*A*). The remaining ∼30% of genes in the shared genomic region either showed expression equivalent to *C. nigoni* wild-type expression (*n*_HIL1_ = 1,124 of 10,473; *n*_HIL2_ = 1,450 of 10,541) or were classified as ambiguous in each HIL (*n*_HIL1_ = 1,913 of 10,473; *n*_HIL2_ = 1,745 of 10,541). We also confirmed that both HILs showed more downregulation overall and an enrichment of downregulated genes on autosomes (fig. 2*B*). These downregulated genes that have lower expression in HILs than in a pure *C. nigoni* genetic background were also enriched for male specific, reproductive, and germline genes (supplementary fig. S3, Supplementary Material online) (Li et al. 2016). In addition, the set of differentially expressed genes (DEGs) in HILs overlapped strongly with one another (fig. 2*C* and *D*). These similarities in differential gene expression patterns across both HILs indicate that their nonoverlapping X-linked introgressions exert similar effects on the transcriptional profiles of genes encoded in the nonintrogressed portion of the *C. nigoni* genome that the HILs have in common. Because the X-chromosome plays a crucial role in dosage compensation and in the regulation of sex-biased genes in nematodes (Meyer 2005; Strome et al. 2014), we explored whether common effects across HILs might result from similar alterations of X-dosage. We analyzed 11 orthologs of *Caenorhabditis elegans* dosage-compensation genes, including female-biased genes that should show very low, if any, expression in pure species males (*sdc-1/2/3*, *dpy-21*, *dpy-26/27/28*, *dpy-30*, and *mix-1*) as well as male-biased genes that should mostly be inactive in females (*xol-1* and *her-1*). However, we could not discern an obvious pattern consistent with a hypothesis of globally disrupted dosage compensation (supplementary fig. S4, Supplementary Material online).

Prior work demonstrated that the X-linked introgressions in these HILs drive changes in posttranscriptional regulation through 22G small RNAs that target spermatogenesis genes to yield greater downregulation on autosomes, ultimately resulting in hybrid male sterility (Li et al. 2016). We next sought to complement these findings by investigating the role of transcriptional regulatory divergence in hybrid male sterility, inspired by the logic of prior work on regulatory divergence inferred with hybrids (Wittkopp et al. 2004; Meiklejohn et al. 2014; Mack and Nachman 2017). Specifically, we aimed to determine the relative incidence of *cis-* and *trans-*acting, as well as nonadditive, regulatory divergence as revealed by perturbed patterns of gene expression caused by the nonoverlapping X-linked introgressions.

### Nonoverlapping Introgressions Produce Similar Patterns of Transgressive Expression

**Fig. 3.**
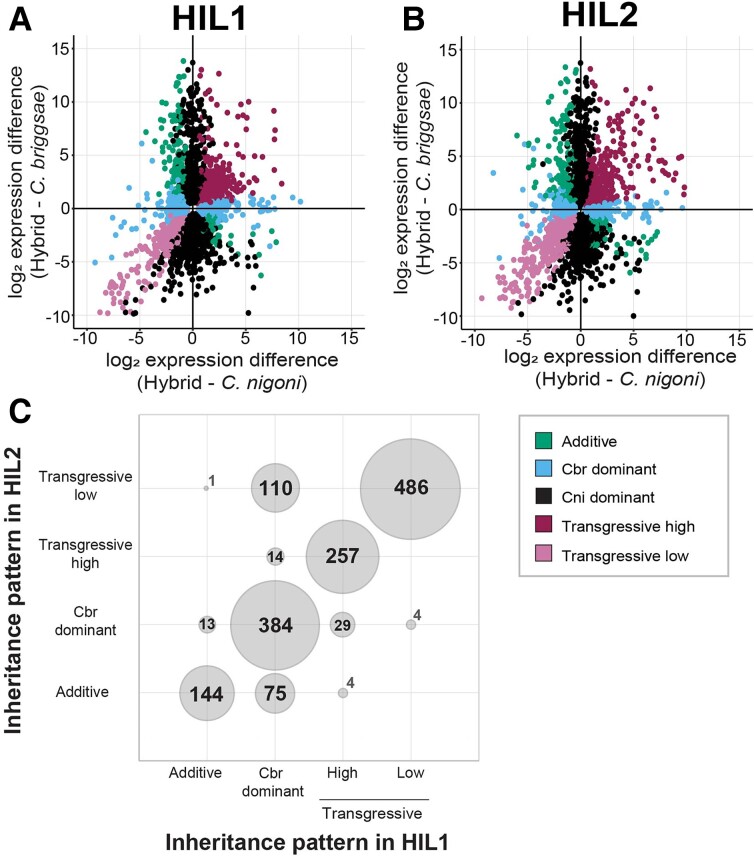
Inheritance pattern of misexpressed genes in hybrid introgression lines. (*A*, *B*) Scatter plots show log_2_ expression differences between each HIL and each parent species with genes colored according to inheritance category, including only those genes in the nonintrogressed portion of the genome. Genes with conserved regulation not shown for clarity. (*C*) Among the 1,521 genes that were differentially expressed in both HILs, 84% corresponded to the same inheritance category in both HILs (*n* = 1,271, points on the diagonal). Overall, 10,361 genes had detectable expression in both HILs (*n*_HIL1_ = 10,361 of 10,473; *n*_HIL2_ = 10,361 of 10,541).

We first explored the additivity of inheritance of differentially expressed genes in hybrids. Based on the three-way comparison of gene expression for each HIL relative to *C. nigoni* and *C. briggsae*, we classified genes into five distinct inheritance categories: 1) *C. nigoni* “dominant” and 2) *C. briggsae* “dominant” genes showed expression levels in the HIL equivalent to one parent species, 3) “additive” genes showed expression in a HIL intermediate between both parents, and 4) “transgressive high” and 5) “transgressive low” genes showed expression in a HIL that exceeded the most extreme expression level of the parental species. Overall, we observed that both HILs showed similar profiles of expression inheritance across the genome (fig. 3*A* and *B*). For the subset of genes that were differentially expressed in both HILs across the shared portion of the genome, 84% were classified in the same inheritance category (*n* = 1,271 of 1,521) (fig. 3*C*). In addition, over half of genes that were downregulated in HILs relative to *C. nigoni* showed transgressive low profiles of expression (*n* = 486 of 827 genes) and over half of upregulated genes exhibited a transgressive high expression profile (*n* = 257 of 444 genes). We observed qualitatively similar patterns when separately considering autosomal genes alone or X-linked genes alone that are encoded outside of the X-linked introgression regions (supplementary table S3, Supplementary Material online). These observations indicate that 1) the X-linked hybrid introgressions lead to a prevailing signature of transgressive low and transgressive high misexpression for genes encoded elsewhere in the genome and 2) this signature affects most of the same sets of genes for both HILs.

### Extensive Overlap of *Cis*- and *Trans*-acting Regulatory Divergence Revealed by Distinct X-Linked Introgressions

**Fig. 4.**
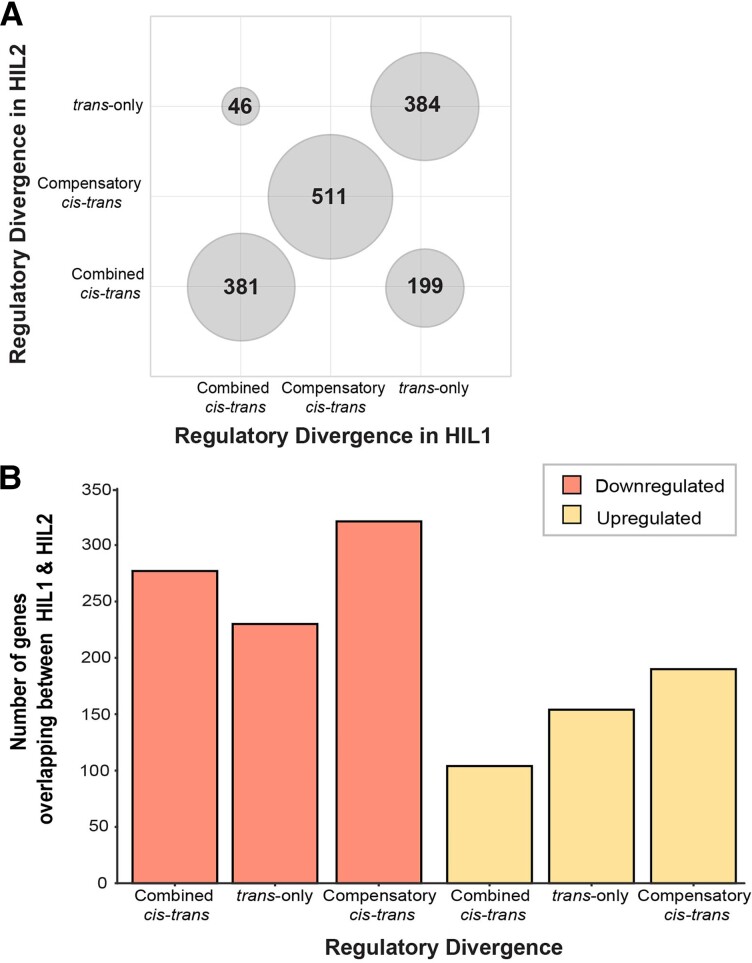
Regulatory divergence underlying misexpression in hybrid introgression lines. (*A*) The regulatory divergence profiles were inferred to be equivalent for 84% of genes that were differentially expressed in both HILs (*n* = 1,276 out of 1,521 commonly differentially expressed genes). ∼35% of these 1,276 DEGs exhibiting the same regulatory divergence profiles (on the diagonal) were upregulated (*n* = 448 out of 1,276 DEGs), and ∼65% of 1,276 DEGs were downregulated and fell into the same regulatory divergence category (*n* = 828 out of 1,276 DEGs). (*B*) Out of the 828 genes downregulated in both HILs and showing the same regulatory divergence profile (orange bars), ∼72% of genes showed evidence of both *cis-* and *trans*-acting regulatory divergence in their expression profiles (*n*_compensatory_ = 321, *n*_*cis-trans*_ = 277, *n*_*trans-*only_ = 230). Out of the 448 genes upregulated in both HILs and exhibiting the same regulatory divergence profile (yellow bars), ∼65% of genes showed evidence of both *cis-* and *trans*-acting regulatory divergence in their expression profiles (*n*_compensatory_ = 190, *n_trans-_*_only_ = 154, *n_cis-trans_* = 104).

We next partitioned genes into distinct categories that define alternative modes of regulatory divergence between gene orthologs of *C. nigoni* and *C. briggsae*. Inspired by the logic used to infer *cis-* and *trans-*regulatory divergence in F1 hybrids (McManus et al. 2010), we used gene expression in HILs relative to their parental species to identify loci showing 1) *trans-*only regulatory divergence, 2) compensatory *cis-* and *trans-*regulatory divergence, and 3) combined *cis-* and *trans-*regulatory divergence (which may be caused by reinforcing and/or compensatory *cis*- and *trans*-acting effects) (fig. 1*C*); the genetic nature of HILs makes *cis*-only regulatory divergence undetectable with these data. Genes inferred to show joint effects of *cis*- and *trans*-acting regulatory divergence could partly reflect differences in relative tissue abundances between HILs and the parental species. Reminiscent of the pattern of nonadditive inheritance, we observed the same regulatory divergence profiles for 84% of the differentially expressed genes (DEGs) that overlapped between the HILs, including both downregulated and upregulated genes (*n* = 1,276 out of 1,521 DEGs) (fig. 4*A*, supplementary tables S4 and S5, Supplementary Material online). Approximately 35% of these 1,276 DEGs that exhibit the same regulatory divergence profiles in both HILs (on the diagonal in fig. 4*A*) were upregulated in the HILs (*n* = 448 out of 1,276 DEGs and *n* = 448 of 516 commonly upregulated genes). The remaining ∼65% of these 1,276 DEGs were downregulated in HILs and come under the same regulatory divergence category (*n* = 828 out of 1,276 DEGs and *n* = 828 of 1,005 commonly downregulated genes).

Among these 1,276 genes, downregulation in the HILs relative to *C. nigoni* was primarily due to divergence in both *cis-* and *trans-*acting factors (both “compensatory” and “combined”), with *trans-*only divergence providing a less frequent source of regulatory divergence (Fisher's exact test *P* < 0.001, fig. 4*B*). In the case of upregulated genes, by contrast, misexpression primarily resulted from compensatory *cis-trans* divergence and *trans-*only regulatory divergence (fig. 4*B*). Additionally, we performed a gene set enrichment analysis across *C. briggsae* and *C. nigoni* for syntenic regions (for each introgression region) using g:Profiler (Raudvere et al. 2019) to identify the Gene Ontology (GO) terms related to RNA-related proteins and transcription factors that differed between the two species, which might suggest differences in *trans-*regulatory mechanisms. However, we found no systematic overabundance of such terms in the syntenic regions corresponding to the introgression regions from either species (supplementary fig. S5, supplementary tables S6 and S7, Supplementary Material online).

These observations indicate that partially distinct regulatory controls might drive the different types of differential gene expression of hybrid introgression lines: *trans-*only divergence is more salient among upregulated genes relative to combined *cis-trans* effects, whereas compensatory *cis-trans* regulatory divergence contributes importantly to genes with both upregulated and downregulated expression in HILs. The extensive overlap in regulatory divergence profiles between the two HILs indicates that these independent introgressions tend to affect the same genes in the same way, ultimately associated with hybrid sterility (i.e., reflecting a common function). Given that a set of regulatory genes that act together toward a common function defines a regulatory module, the perturbation of the same genes in the same way by the two nonoverlapping X-linked introgressions provides evidence for a shared regulatory module underlying misregulation and misexpression of genes in hybrids that associates with hybrid male sterility in this system.

## Discussion

We aimed to decipher the role that gene regulatory divergence might play as a mechanism mediating X-autosome interactions that, in turn, could contribute to hybrid male sterility in *Caenorhabditis* nematodes. Using the gene expression data of two hybrid introgression lines (Li et al. 2016), we identified hundreds of genes showing distinct classes of nonadditive inheritance and regulatory divergence in hybrids. High overlap across both HILs for differentially expressed genes, sets of transgressive genes, and contributions to *cis*- and *trans*-regulatory divergence together suggest that the genetically nonoverlapping X-linked introgressions disrupt the transcriptome in similar ways in these hybrids, predominantly due to the joint effects of *cis*- and *trans*-acting regulatory divergence that exert compensatory effects on gene expression.

### Regulatory Divergence and Speciation

Conceptual arguments and empirical evidence support the idea that *cis*-acting regulatory divergence between species is common, likely facilitated by only small pleiotropic effects of mutations to *cis*-regulatory elements (Stern 2000; Prud’homme et al. 2007; Wray 2007; Mack and Nachman 2017). Our analysis of homozygous hybrid introgression lines, however, is blind to detection of *cis*-only differences, letting us focus on the incidence of *trans*-only and joint *cis*- and *trans*-acting regulatory divergence. We documented how divergence in both *cis-* and *trans*-acting factors that affect a given gene's expression provides a common source of misexpression in hybrid introgression lines, outnumbering *trans*-only effects by two-to-one. These observations contribute to growing support for a substantial contribution of the combined influence of *cis*- and *trans*-acting regulatory divergence affecting a given gene's expression, as revealed in interspecies hybrids (Landry et al. 2005; Takahasi et al. 2011; Goncalves et al. 2012; Verta et al. 2016; Mack and Nachman 2017; Metzger et al. 2017; Sánchez-Ramírez et al. 2021).

Differences in the reproductive modes of *C. nigoni* and *C. briggsae* might also lead to differences in the overall genomic composition of the two species. Gonochoristic *C. nigoni* has a larger genome (∼136 Mb) compared with the hermaphroditic *C. briggsae* (108 Mb), attributed to the loss of repetitive DNA sequences and male-biased genes in *C. briggsae*, such that ∼6,000 genes lack orthologs between them and are unique to one species or the other (Ren et al. 2018; Yin et al. 2018; Cutter 2019). Such changes in gene content as well as structural differences involving homologous and nonhomologous chromosomes (Bi et al. 2015; Ren et al. 2018; Yin et al. 2018) may alter genetic networks and the mechanisms of gene regulation between species, including changes associated with either loss or gain of orthologous genes or gene regulators with species-specific effects. Such species-specific regulation could be sensitive to the X-linked introgressions to provide a source of misexpression in hybrids. Because our analysis focuses on 1:1 orthologs, inference of *trans*-acting regulatory divergence may partly reflect such divergence of genes without detectable 1:1 orthologs.

The regulation of gene expression involves interactions between loci. Consequently, disruption of gene regulation from the joint influence of *cis*- and *trans*-acting factors can present itself as Dobzhansky–Muller expression incompatibilities if they cause fitness deficits in hybrids and thus can be a mechanism underlying postzygotic reproductive isolation (Mack and Nachman 2017). The finding that compensatory regulatory evolution between species is pervasive, as also demonstrated in this study, provides support for the idea that regulatory divergence can contribute to reproductive isolation as an important source of hybrid incompatibility (Meiklejohn et al. 2014; Mack and Nachman 2017; Signor and Nuzhdin 2018).

### Stabilizing Selection on Overall Expression Despite Molecular Evolution of Gene Regulation

Stabilizing selection represents the prevailing selective force on gene expression evolution and, in particular, underlies the widespread pattern of conservation in expression of orthologous genes between species (Gilad et al. 2006). Stabilizing selection on the expression level as a phenotype, however, can reflect different underlying mechanisms at the molecular level. They include the selective elimination of deleterious mutations by purifying selection on *cis*-regulatory elements as well as compensatory evolution that involves distinct regulatory changes that exert counteracting increasing and decreasing effects that jointly act to maintain overall expression levels (Mack and Nachman 2017; Signor and Nuzhdin 2018).

Our analysis provides evidence for the latter mechanism of compensatory regulatory evolution as an important molecular incarnation of stabilizing selection. We show that compensatory *cis-trans* regulatory divergence is a common feature of misexpression in hybrids, implicating stabilizing selection via compensatory regulatory divergence in the conservation of gene expression levels between species (Lemos et al. 2005; Coolon et al. 2014; Hodgins-Davis et al. 2015; Signor and Nuzhdin 2018). Such compensatory changes can arise specifically via serial changes to *cis*- and *trans-*acting regulatory factors in an evolutionary feedback within gene regulatory networks that act to reduce the deleterious pleiotropic side effects of mutations elsewhere (e.g., modifying negatively pleiotropic side effects of a net beneficial mutation) (Maisnier-Patin and Andersson 2004; Angst and Hall 2013; Wang et al. 2015; Signor and Nuzhdin 2018). For example, *cis*-acting changes can lead to expression divergence in a number of ways: changes in core promoter sequence and enhancers leading to direct effects on transcript abundance, changes in transcription factor binding sites altering the *cis*-regulatory activity, and changes in chromatin accessibility and posttranscriptional processes that ultimately affect mRNA abundances (Hill et al. 2021). *Trans*-acting changes, on the other hand, can be due to *cis*-acting mutations in coding and noncoding sequences of the genes producing the *trans*-factor (Hill et al. 2021). Alterations of cofactors, chromatin regulators, and other metabolic genes that form part of the gene regulatory network can also lead to *trans*-acting changes (Hill et al. 2021).

Our results add to the growing literature in support of the idea that stabilizing selection operates as a major selective regime on gene expression and that it can yield interspecies expression similarity in spite of profound molecular evolution in gene regulatory controls (Landry et al. 2005; Gilad et al. 2006; Takahasi et al. 2011; Goncalves et al. 2012; Wang et al. 2015; Verta et al. 2016; Metzger et al. 2017; Sánchez-Ramírez et al. 2021). More broadly, such pervasive compensatory *cis-trans* regulatory divergence represents a key evolutionary agent for developmental system drift and as a potential source of Dobzhansky–Muller incompatibilities (True and Haag 2001; Mack and Nachman 2017; Cutter and Bundus 2020). Future work in this area, however, will benefit from considering regulatory divergence for expression when explicitly measured in distinct tissues or in specific cells to better characterize how hybrid tissue allometry may intersect with differential expression.

### Multilocus Expression Incompatibilities in Hybrid Male Sterility

The spermatogenesis process in nematode males, which is often disrupted in interspecies hybrids, involves an interconnected network of genes with important interactions between autosomal and X-linked loci (Li et al. 2016; Bundus et al. 2018). Our results, and those of Li et al. (2016), show that distinct introgression segments on the X-chromosome disrupt in similar ways the transcriptome of autosomally encoded genes. By affecting the same spermatogenesis-related genes on the autosomes, a broad region of the X-chromosome is capable of perturbing proper spermatogenesis to cause infertility (Li et al. 2016). This overlapping sensitivity of the autosomal transcriptome to different X-linked genetic perturbations implicates the presence of multiway negative genetic interactions as a complex form of Dobzhansky–Muller expression incompatibility.

Evolution in partially redundant gene regulatory networks (GRN) can lead to compensatory molecular evolutionary changes that nonetheless retain a constant phenotype, termed developmental system drift (True and Haag 2001; Cutter and Bundus 2020; Schiffman and Ralph 2022). Consequently, as different mutations accumulate independently in separate species, developmental system drift can lead their molecular genetic pathways to trace distinct trajectories of connectivity, despite reaching the same phenotype. In hybrids, however, the crosstalk between divergent pathways can lead to genetic mismatches. Such mismatches can disrupt epistatic interactions between the coevolved expression of loci in the GRN, a form of Dobzhansky–Muller incompatibility that may lead to hybrid dysfunction (Tulchinsky et al. 2014; Satokangas et al. 2020). Because *cis-* and *trans-*acting regulatory controls within a GRN jointly serve to mediate multilocus epistatic interactions through pairwise or higher order complexes, such regulatory divergence can influence the likelihood that genetic incompatibilities arise between species and contribute to reproductive isolation (Satokangas et al. 2020).

Simple pairwise interactions that involve a given locus, however, should not be influenced directly by multiple distinct genetic perturbations. We found that similar disruptions to the transcriptome emerge in hybrid introgression lines due to distinct, nonoverlapping genetic perturbations of the X-chromosome, often mediated by compensatory *cis*- and *trans*-acting regulatory divergence. Consequently, these observations require the existence of multiway incompatibility interactions that involve at least three, but perhaps many more, genes (Burkart-Waco et al. 2012). Thus, the GRN responsible for male fertility in this system may be structured in a way that makes it susceptible to disruption in hybrids as a result of multiway incompatibilities. Given that the evolutionary dynamics of multiway DMIs differ from pairwise DMIs (Orr 1995; Kondrashov 2003), it remains an important outstanding question to determine whether regulatory divergence in GRNs is predisposed more generally to producing multiway DMIs. Such differences among the GRNs that control the development of different traits might help explain why some aspects of development are more prone to manifesting DMIs and hybrid dysfunction (Cutter and Bundus 2020). If GRNs that control the developmental programs responsible for male fertility are disproportionately prone to multiway DMIs, then, such genetic architectures might provide a mechanistic rationale for the “fragile male” hypothesis to explain Haldane's rule (Wu and Davis 1993; Cutter 2018).

### Methodological Constraints and Future Recommendations

Our study connects gene regulation and speciation by focusing on regulatory divergence as a mechanism underlying gene misexpression in sterile hybrid male nematodes. Specifically, we observed extensive transgressive expression of autosomal genes due to divergence involving the compensatory and joint effects of *cis-* and *trans*-acting factors. However, we could not distinguish the role of *cis*-only divergence with these homozygous introgression lines, and our analysis only tested the role of two X-linked regions out of the potentially multiple incompatible regions across the genome. Nonetheless, our results reinforce conclusions from previous work in this system and expand the literature that links regulatory divergence to speciation (Li et al. 2016; Sánchez-Ramírez et al. 2021). Gene regulation in *C. nigoni* is more robust to introgression from *C. briggsae* than vice versa (Xie et al. 2022). Given these observations, we further highlight that our study can, additionally, be seen as a proof of concept for using a large library of introgression lines to gain insights into regulatory divergence as a mechanism of hybrid male sterility.

An important caveat to our inferences is that differences in gene expression between hybrids and parental species may in part reflect tissue allometric differences resulting from dysfunctional gonad development in hybrids. The gonads of hybrids suffer from morphological defects, making them smaller than the wild-type gonads (Woodruff et al. 2010). Smaller gonads and fewer germline cells could potentially result in the observed downregulation of spermatogenesis-related genes (Deng et al. 2011). However, it is unlikely that this is the sole reason for the observed misexpression in the hybrids. These developmental defects produce a range of phenotypes, including partially formed gonads, possibly retaining germline cells in the pooled samples, albeit less than the wild type (Woodruff et al. 2010). Additionally, it is unclear whether this is due to fewer cells or to smaller cells (Woodruff et al. 2010; Sánchez-Ramírez et al. 2021). However, since we have data only for whole bodies, we could not disentangle possible differences in cell composition. Given that this gonadal developmental defect is common to both hybrid introgression lines, however, comparison of the two HILs to one another is unlikely to be highly sensitive to this potential issue of gonad versus somatic tissue contributions. Furthermore, evidence for the misexpression of genes in interspecies hybrids is consistent across different taxa, and studies have shown downregulation of spermatogenesis and germline genes underlying hybrid male sterility using both whole-body and testis-only samples (Artieri et al. 2007; Moehring et al. 2007; Catron and Noor 2008; Sundararajan and Civetta 2011; Li et al. 2016; Mack et al. 2016; Banho et al. 2021; Xie et al. 2022).

With these constraints in mind, future studies will benefit from direct consideration or elimination of possible influence from allometric tissue differences. For example, such analysis could compare tissue- or cell-specific samples with whole-body samples (Deng et al. 2011; Sundararajan and Civetta 2011; Kopania et al. 2022). In addition to allele-specific expression analysis using F1 hybrids, studies can leverage biological collections of hybrid introgression lines to identify incompatible regions of the genome and their consequence on the transcriptome. Comparisons between X-linked and autosomal introgressions as well as between sterile and fertile hybrid introgression lines can be used to further understand the relative roles of different incompatible regions in causing misexpression in hybrids (Ferguson et al. 2013; Bi et al. 2015; Guerrero et al. 2016; Li et al. 2016; Xie et al. 2022). Studies can also span across different developmental stages to understand the temporal effects of gene regulatory divergence, as well as use gene expression analyses beyond the F1 hybrid generation, to more fully understand the role of gene regulation in speciation.

## Methods

### Data Set

We accessed the mRNA sequence data set produced by Li et al. (2016) from NCBI (SRP067756). This data set consists of ∼8 million high-quality paired-end (2 × 150 bp) Illumina MiSeq reads per sample from pools of 300 young adult males, with replication in triplicate for each of *C. nigoni* (JU1421), *C. briggsae* (AF16), ZZY10330 (hybrid introgression line 1), and ZZY10307 (hybrid introgression line 2) (Li et al. 2016). Li et al. obtained young adult hybrid males by crossing fertile hybrid females carrying introgression with *C. nigoni* males and used the presence of green fluorescence from GFP to identify hybrid males with the X-linked introgression (Li et al. 2016). The genomes of hybrid introgression lines (HILs) primarily carry *C. nigoni* DNA, with each HIL containing an independent, nonoverlapping fragment from the *C. briggsae* X-chromosome that contributes to hybrid male sterility (Bi et al. 2015; Li et al. 2016). In *Caenorhabditis* males, the X-chromosome is hemizygous due to the XO sex determination system. Based on our analysis of mapped reads below, we affirmed that the right arm of the X-chromosome in ZZY10330 (HIL1) carries a ∼4.8 Mb fragment from *C. briggsae* containing a total of 311 orthologous genes with detectable expression inside the fragment (out of a total of 470 annotated orthologous genes) and 11,097 autosomal and X-linked orthologous genes with detectable expression outside the introgression from the *C. nigoni* genomic background (from a total of 13,505 annotated orthologous genes outside the introgressed region). The ZZY10307 (HIL2) strain has an introgression fragment of size ∼7.4 Mb in the middle of the X-chromosome with a total of 631 orthologous genes inside the introgression (out of a total of 867 annotated orthologous genes) and 10,852 outside the introgression with detectable expression.

### Analysis of mRNA-Seq Reads

We mapped the same mRNA reads separately against both the *C. nigoni* (JU1422) reference genome “nigoni.pc_2016.07.14” (Yin et al. 2018) and the *C. briggsae* (AF16) reference genome “CB4” (Ross et al. 2011) using STAR with default parameters (Dobin et al. 2013). To identify and confirm the introgression boundaries on the X-chromosome, for each HIL, we compared read counts mapped to *C. briggsae* and *C. nigoni* reference genomes to determine which genes mapped better to the *C. briggsae* genome as a consequence of ∼21% synonymous-site sequence divergence of *C. nigoni* orthologous loci (Thomas et al. 2015). For each HIL, we calculated and plotted the difference in log_2_(mean read count + 0.1) when mapped to the *C. briggsae* reference genome versus the *C. nigoni* reference genome for each gene on the X-chromosome (supplementary figs. S1 and S2 and tables S1 and S2, Supplementary Material online). Genes with positive values for this difference indicated better mapping to the *C. briggsae* reference genome, clustering at the regions expected to contain the introgression which we then used to define the introgression boundaries for each HIL. In this way, we confirmed the presence of a ∼4.8 Mb fragment introgressed on the right arm of the X-chromosome in HIL1 from *C. briggsae* (*C. briggsae* positions 16,392,964 bp to 21,277,039 bp, defined by the genes WBGene00041261 on the left and WBGene00031970 on the right of the fragment) (supplementary table S1, Supplementary Material online) and a ∼7.4 Mb fragment in the middle of the X-chromosome of HIL2 (*C. briggsae* positions 4,742,872 bp to 12,165,712 bp, defined by the genes WBGene00036950 on the left and WBGene00032130 on the right of the introgressed fragment) (fig. 1*A*; supplementary table S2, Supplementary Material online).

To quantify gene expression appropriately for genes inside and outside the introgression region, we considered mapped reads to the *C. nigoni* reference genome for those genes determined to be outside the introgression region of a given HIL, and mapped reads to the *C. briggsae* reference genome for those genes inside each introgression. We quantified gene expression of all the genes using FeatureCounts (Liao et al. 2014) and restricted subsequent analysis of the read count data to overlap with the set of 13,975 1:1 orthologous genes defined in Sánchez-Ramírez et al. (2021). Differential gene expression analysis was performed for orthologous genes using DESeq2 (Love et al. 2014) in R Studio (RStudio version 4.1.2). To perform our analysis across different species and hybrid introgression lines, we provided the data for the respective pairwise comparison and defined the variable “∼species” in our design formula. “∼species” contained the species information for each sample, that is, *C. briggsae*, *C. nigoni*, HIL1, and HIL2. We used the default method of DESeq2 for multiple test correction which is the Benjamini–Hochberg false discovery rate. Genes showing Benjamini–Hochberg false discovery rate-adjusted *P*_adj_ < 0.05 between *C. nigoni* and a given HIL were considered to be differentially expressed. We then categorized genes from their expression profiles to infer additivity of inheritance pattern and *cis–trans*-regulatory divergence, similar to McManus et al. (2010). Genes were classified into five distinct inheritance categories based on their expression relative to the wild-type expression of both *C. briggsae* and *C. nigoni*: 1) and 2) genes showing expression levels in the HIL equivalent to one of the parent species were classified as *C. nigoni* (or *C. briggsae)* “dominant,” 3) genes showing expression in a HIL significantly different from and intermediate between both parents were termed “additive,” and 4) and 5) genes showing expression in a HIL that was significantly above or below both parental species were classified as genes with “transgressive high” or “transgressive low” expression, respectively (fig. 1*B*).

To infer distinct kinds of regulatory divergence, we classified genes according to distinct profiles of differential expression in pairwise contrasts between each of *C. briggsae*, *C. nigoni*, and hybrid introgression lines. In particular, we inferred genes to exhibit 1) *trans-*only regulatory divergence when *C. nigoni* expression differed significantly from *C. briggsae* and the HIL in the absence of significant differential expression of the HIL from *C. briggsae*, 2) compensatory *cis–trans*-regulatory divergence when the HIL showed significant differential expression with both *C. nigoni* and *C. briggsae* despite *C. nigoni* and *C. briggsae* displaying no significant difference between one another, 3) combined *cis-* and *trans-*regulatory divergence when all three pairwise contrasts displayed significant expression differences (because this category includes genes in HILs showing expression higher or lower than both parents as well as genes showing intermediate expression between parents, the *cis–trans* effects could be either compensatory or reinforcing), 4) no effect when genes in HIL showed no significant differential expression with *C. nigoni* despite significant difference in expression *C. nigoni* and *C. briggsae* and *C. briggsae* and HIL, 5) conserved regulation when the gene showed no significant differential expression in any pairwise contrast, or 6) ambiguous regulatory divergence for all other expression circumstances (fig. 1*C*). It is important to note that although our logic for classification is based on McManus et al. (2010) and Meiklejohn et al. (2014) that exploited heterozygous autosomal introgressions in *Drosophila*, our criteria differ to account for the genomic architecture of hemizygous X-linked introgressions of hybrid males in this study, rather than allele-specific expression of F1 hybrids. From previous work, we know that hybrid males in this system suffer from gonad developmental defects which can contribute to differences in cellular composition and gene expression (Li et al. 2016 and Sánchez-Ramírez et al. 2021). The use of expression data of whole organisms in our analysis thus limited our ability to explicitly control for the differences in cellular composition between hybrids and parental samples, which is a caveat to our analysis. However, the general patterns across the two HILs likely remain the same as this gonadal defect is common to both hybrids.

## Supplementary Material

evad055_Supplementary_Data

## Data Availability

No new data were generated in support of this work. The mRNA sequencing data underlying this article are available in NCBI Gene Expression Omnibus at GEO; http://www.ncbi.nlm.nih.gov/geo/ and can be accessed with accession number GSE76306. The *C. nigoni* and *C. briggsae* reference genomes used in this study can be accessed under the NCBI BioProject accessions PRJNA384657 and PRJNA10731, respectively.

## References

[evad055-B1] Angst DC , Hall AR. 2013. The cost of antibiotic resistance depends on evolutionary history in *Escherichia coli*. BMC Evol Biol. 13:1–8. 23914906 10.1186/1471-2148-13-163 PMC3751127

[evad055-B2] Artieri CG , Haerty W, Singh RS. 2007. Association between levels of coding sequence divergence and gene misregulation in Drosophila male hybrids. J Mol Evol. 65:697–704. 18026889 10.1007/s00239-007-9048-2

[evad055-B3] Baird SE , Seibert SR. 2013. Reproductive isolation in the Elegans-group of Caenorhabditis. Nat Sci. 5(4A):18.

[evad055-B4] Banho CA , Mérel V, Oliveira TY, Carareto C, Vieira C. 2021. Comparative transcriptomics between Drosophila mojavensis and D. arizonae reveals transgressive gene expression and underexpression of spermatogenesis-related genes in hybrid testes. Sci Rep. 11(1):1–5. 33972659 10.1038/s41598-021-89366-2 PMC8110761

[evad055-B5] Barrière A , Ruvinsky I. 2014. Pervasive divergence of transcriptional gene regulation in *Caenorhabditis* nematodes. PLoS Genet. 10:e1004435. 10.1371/journal.pgen.1004435 PMC4072541 24968346

[evad055-B6] Bedford T , Hartl DL. 2009. Optimization of gene expression by natural selection. Proc Natl Acad Sci U S A. 106(4):1133–1138. 19139403 10.1073/pnas.0812009106 PMC2633540

[evad055-B7] Bell GDM , Kane NC, Rieseberg LH, Adams KL. 2013. RNA-Seq analysis of allele-specific expression, hybrid effects, and regulatory divergence in hybrids compared with their parents from natural populations. Genome Biol Evol. 5:1309–1323. 23677938 10.1093/gbe/evt072 PMC3730339

[evad055-B8] Bi Y , et al 2015. A genome-wide hybrid incompatibility landscape between *Caenorhabditis briggsae* and *C. nigoni*. PLoS Genet. 11:e1004993. 10.1371/journal.pgen.1004993 PMC4334894 25692300

[evad055-B9] Bi Y , et al 2019. Specific interactions between autosome and X chromosomes cause hybrid male sterility in Caenorhabditis species. Genetics 212(3):801–813. 31064822 10.1534/genetics.119.302202 PMC6614896

[evad055-B10] Bundus JD , Alaei R, Cutter AD. 2015. Gametic selection, developmental trajectories, and extrinsic heterogeneity in Haldane's rule. Evolution 69(8):2005–2017. 26102479 10.1111/evo.12708

[evad055-B11] Bundus JD , Wang D, Cutter AD. 2018. Genetic basis to hybrid inviability is more complex than hybrid male sterility in *Caenorhabditis* nematodes. Heredity (Edinb). 121:169–182. 29626207 10.1038/s41437-018-0069-y PMC6039526

[evad055-B12] Burkart-Waco D , et al 2012. Hybrid incompatibility in *Arabidopsis* is determined by a multiple-locus genetic network. Plant Physiol. 158:801–812. 22135429 10.1104/pp.111.188706 PMC3271768

[evad055-B13] Catron DJ , Noor MAF. 2008. Gene expression disruptions of organism versus organ in Drosophila species hybrids. PLoS One. 3:e3009. 10.1371/journal.pone.0003009 PMC2500191 18714377

[evad055-B14] Coolon JD , et al 2015. Molecular mechanisms and evolutionary processes contributing to accelerated divergence of gene expression on the *Drosophila* X chromosome. Mol Biol Evol. 32:2605–2615. 26041937 10.1093/molbev/msv135 PMC4592344

[evad055-B15] Coolon JD , McManus CJ, Stevenson KR, Graveley BR, Wittkopp PJ. 2014. Tempo and mode of regulatory evolution in *Drosophila*. Genome Res. 24:797–808. 24567308 10.1101/gr.163014.113 PMC4009609

[evad055-B16] Coughlan JM , Matute DR. 2020. The importance of intrinsic postzygotic barriers throughout the speciation process. Philos Trans R Soc Lond B Biol Sci. 375:20190533. 10.1098/rstb.2019.0533 PMC7423280 32654642

[evad055-B17] Coyne JA , Orr HA. 1998. The evolutionary genetics of speciation.Philos Trans R Soc Lond B Biol Sci. 353:287–305. 9533126 10.1098/rstb.1998.0210 PMC1692208

[evad055-B18] Coyne JA , Orr HA. 2004. Speciation. Sunderland, MA: Sinauer Associates Sunderland.

[evad055-B19] Cutter AD . 2018. X exceptionalism in Caenorhabditis speciation. Mol Ecol. 27:3925–3934. 29134711 10.1111/mec.14423

[evad055-B20] Cutter AD . 2019. Reproductive transitions in plants and animals: selfing syndrome, sexual selection and speciation. New Phytol. 224(3):1080–1094. 31336389 10.1111/nph.16075

[evad055-B21] Cutter AD , Bundus JD. 2020. Speciation and the developmental alarm clock. Elife 9:e56276. 10.7554/eLife.56276 PMC7481004 32902377

[evad055-B22] Cutter AD , Choi JY. 2010. Natural selection shapes nucleotide polymorphism across the genome of the nematode Caenorhabditis briggsae. Genome Res. 20(8):1103–1111. 20508143 10.1101/gr.104331.109 PMC2909573

[evad055-B23] Delph LF , Demuth JP. 2016. Haldane's rule: genetic bases and their empirical support. J Hered. 107:383–391. 27233288 10.1093/jhered/esw026

[evad055-B24] Deng X , et al 2011. Evidence for compensatory upregulation of expressed X-linked genes in mammals, Caenorhabditis elegans and Drosophila melanogaster. Nat Genet. 43(12):1179–1185. 22019781 10.1038/ng.948 PMC3576853

[evad055-B25] Dobin A , et al 2013. STAR: ultrafast universal RNA-seq aligner. Bioinformatics 29:15–21. 23104886 10.1093/bioinformatics/bts635 PMC3530905

[evad055-B26] Felix MA , Braendle C, Cutter AD. 2014. A streamlined system for species diagnosis in Caenorhabditis (Nematoda: Rhabditidae) with name designations for 15 distinct biological species. PLoS One. 9(4):e94723. 10.1371/journal.pone.0094723 PMC3984244 24727800

[evad055-B27] Ferguson J , Gomes S, Civetta A. 2013. Rapid male-specific regulatory divergence and down regulation of spermatogenesis genes in *Drosophila* species hybrids. PLoS One. 8:e61575. 10.1371/journal.pone.0061575 PMC3623997 23593487

[evad055-B28] Gilad Y , Oshlack A, Rifkin SA. 2006. Natural selection on gene expression. Trends Genet. 22:456–461. 16806568 10.1016/j.tig.2006.06.002

[evad055-B29] Gomes S , Civetta A. 2015. Hybrid male sterility and genome-wide misexpression of male reproductive proteases. Sci Rep. 5:11976. 10.1038/srep11976 PMC4491705 26146165

[evad055-B30] Goncalves A , et al 2012. Extensive compensatory *cis*-*trans* regulation in the evolution of mouse gene expression. Genome Res. 22:2376–2384. 22919075 10.1101/gr.142281.112 PMC3514667

[evad055-B31] Graze RM , et al 2014. What the X has to do with it: differences in regulatory variability between the sexes in *Drosophila simulans*. Genome Biol Evol. 6:818–829. 24696400 10.1093/gbe/evu060 PMC4007535

[evad055-B32] Guerrero RF , Posto AL, Moyle LC, Hahn MW. 2016. Genome-wide patterns of regulatory divergence revealed by introgression lines. Evolution 70(3):696–706. 26842879 10.1111/evo.12875

[evad055-B33] Gupta BP , Johnsen R, Chen N. 2007. Genomics and biology of the nematode Caenorhabditis briggsae. WormBook. 1–16. doi:10.1895/wormbook.1.136.1 PMC4781269 18050493

[evad055-B34] Haag ES . 2005. The evolution of nematode sex determination: C. elegans as a reference point for comparative biology. WormBook. 1–14. doi:10.1895/Wormbook.1.120.1 PMC4781019 18050417

[evad055-B35] Haldane JB . 1922. Sex ratio and unisexual sterility in hybrid animals. J Genet. 12:101–109.

[evad055-B36] Hill MS , Vande Zande P, Wittkopp PJ. 2021. Molecular and evolutionary processes generating variation in gene expression. Nat Rev Genet. 22(4):203–215. 33268840 10.1038/s41576-020-00304-w PMC7981258

[evad055-B37] Hodgins-Davis A , Rice DP, Townsend JP. 2015. Gene expression evolves under a house-of-cards model of stabilizing selection. Mol Biol Evol. 32:2130–2140. 25901014 10.1093/molbev/msv094 PMC4592357

[evad055-B38] Johnson NA , Porter AH. 2000. Rapid speciation via parallel, directional selection on regulatory genetic pathways. J Theor Biol. 205(4):527–542. 10931750 10.1006/jtbi.2000.2070

[evad055-B39] Kondrashov AS . 2003. Accumulation of Dobzhansky–Muller incompatibilities within a spatially structured population. Evolution. 57:151–153. 12643575 10.1111/j.0014-3820.2003.tb00223.x

[evad055-B40] Kopania EE , Larson EL, Callahan C, Keeble S, Good JM. 2022. Molecular evolution across mouse spermatogenesis. Mol Biol Evol. 39(2):msac023. 10.1093/molbev/msac023 PMC8844503 35099536

[evad055-B41] Kozlowska JL , Ahmad AR, Jahesh E, Cutter AD. 2012. Genetic variation for postzygotic reproductive isolation between Caenorhabditis briggsae and Caenorhabditis sp. 9. Evolution 66(4):1180–1195. 22486697 10.1111/j.1558-5646.2011.01514.x

[evad055-B42] Landry CR , et al 2005. Compensatory *cis-trans* evolution and the dysregulation of gene expression in interspecific hybrids of *Drosophila*. Genetics 171:1813–1822. 16143608 10.1534/genetics.105.047449 PMC1456106

[evad055-B43] Landry CR , Hartl DL, Ranz JM. 2007. Genome clashes in hybrids: insights from gene expression. Heredity (Edinb). 99:483–493. 17687247 10.1038/sj.hdy.6801045

[evad055-B44] Lemos B , Meiklejohn CD, Cáceres M, Hartl DL. 2005. Rates of divergence in gene expression profiles of primates, mice, and flies: stabilizing selection and variability among functional categories. Evolution 59:126–137. 15792233

[evad055-B45] Li R , et al 2016. Specific down-regulation of spermatogenesis genes targeted by 22G RNAs in hybrid sterile males associated with an X-Chromosome introgression. Genome Res. 26:1219–1232. 27197225 10.1101/gr.204479.116 PMC5052035

[evad055-B46] Li XC , Fay JC. 2017. *Cis*-regulatory divergence in gene expression between two thermally divergent yeast species. Genome Biol Evol. 9:1120–1129. 28431042 10.1093/gbe/evx072 PMC5554586

[evad055-B47] Liao Y , Smyth GK, Shi W. 2014. Featurecounts: an efficient general-purpose read summarization program. Bioinformatics 30:923–930. 24227677 10.1093/bioinformatics/btt656

[evad055-B48] Llopart A . 2012. The rapid evolution of X-linked male-biased gene expression and the large-X effect in *Drosophila yakuba*, *D. santomea*, and their hybrids. Mol Biol Evol. 29:3873–3886. 22844069 10.1093/molbev/mss190

[evad055-B49] Love MI , Huber W, Anders S. 2014. Moderated estimation of fold change and dispersion for RNA-Seq data with DESeq2. Genome Biol. 15:1–21. 10.1186/s13059-014-0550-8 PMC4302049 25516281

[evad055-B50] Lu X , et al 2010. Genome-wide misexpression of X-linked versus autosomal genes associated with hybrid male sterility. Genome Res. 20:1097–1102. 20511493 10.1101/gr.076620.108 PMC2909572

[evad055-B51] Mack KL , Campbell P, Nachman MW. 2016. Gene regulation and speciation in house mice. Genome Res. 26:451–461. 26833790 10.1101/gr.195743.115 PMC4817769

[evad055-B52] Mack KL , Nachman MW. 2017. Gene regulation and speciation. Trends Genet. 33:68–80. 27914620 10.1016/j.tig.2016.11.003 PMC5182078

[evad055-B53] Maheshwari S , Barbash DA. 2011. The genetics of hybrid incompatibilities. Annu Rev Genet. 45:331–355. 21910629 10.1146/annurev-genet-110410-132514

[evad055-B54] Maisnier-Patin S , Andersson DI. 2004. Adaptation to the deleterious effects of antimicrobial drug resistance mutations by compensatory evolution. Res Microbiol. 155:360–369. 15207868 10.1016/j.resmic.2004.01.019

[evad055-B55] McManus CJ , et al 2010. Regulatory divergence in *Drosophila* revealed by mRNA-Seq. Genome Res. 20:816–825. 20354124 10.1101/gr.102491.109 PMC2877578

[evad055-B56] Meiklejohn CD , Coolon JD, Hartl DL, Wittkopp PJ. 2014. The roles of *cis* - and *trans* - regulation in the evolution of regulatory incompatibilities and sexually dimorphic gene expression. Genome Res. 24:84–95. 24043293 10.1101/gr.156414.113 PMC3875864

[evad055-B57] Metzger BPH , Wittkopp PJ, Coolon JD. 2017. Evolutionary dynamics of regulatory changes underlying gene expression divergence among *Saccharomyces* species. Genome Biol Evol. 9:843–854. 28338820 10.1093/gbe/evx035 PMC5604594

[evad055-B58] Meyer BJ . 2005. X-Chromosome dosage compensation. WormBook:1–14. 10.1895/wormbook.1.8.1 PMC4781388 18050416

[evad055-B59] Michalak P . 2003. Genome-wide patterns of expression in *Drosophila* pure species and hybrid males. Mol Biol Evol. 20:1070–1076. 12777520 10.1093/molbev/msg119

[evad055-B60] Moehring AJ , Teeter KC, Noor MAF. 2007. Genome-wide patterns of expression in Drosophila pure species and hybrid males. II. Examination of multiple-species hybridizations, platforms, and life cycle stages. Mol Biol Evol. 24(1):137–145. 17032727 10.1093/molbev/msl142

[evad055-B61] Oka A , Shiroishi T. 2014. Regulatory divergence of X-linked genes and hybrid male sterility in mice. Genes Genet Syst. 89:99–108. 25475933 10.1266/ggs.89.99

[evad055-B62] Orr HA . 1995. The population genetics of speciation: the evolution of hybrid incompatibilities. Genetics 139:1805–1813. 7789779 10.1093/genetics/139.4.1805 PMC1206504

[evad055-B63] Presgraves DC . 2010. Darwin and the origin of interspecific genetic incompatibilities. Am Nat. 176(Suppl 1):S45–S60. 21043780 10.1086/657058

[evad055-B64] Prud’homme B , Gompel N, Carroll SB. 2007. Emerging principles of regulatory evolution. Proc Natl Acad Sci U S A. 104(Suppl 1):8605–8612. 17494759 10.1073/pnas.0700488104 PMC1876436

[evad055-B65] Ranz JM , Namgyal K, Gibson G, Hartl DL. 2004. Anomalies in the expression profile of interspecific hybrids of *Drosophila melanogaster* and *Drosophila simulans*. Genome Res. 14:373–379. 14962989 10.1101/gr.2019804 PMC353219

[evad055-B66] Raudvere U , et al 2019. G: Profiler: a web server for functional enrichment analysis and conversions of gene lists (2019 update). Nucleic Acids Res. 47(W1):W191–W198. 31066453 10.1093/nar/gkz369 PMC6602461

[evad055-B67] Ren X , et al 2018. Genomic basis of recombination suppression in the hybrid between Caenorhabditis briggsae and C. nigoni. Nucleic Acids Res. 46(3):1295–1307. 29325078 10.1093/nar/gkx1277 PMC5814819

[evad055-B68] Ross JA , et al 2011. *Caenorhabditis briggsae* recombinant inbred line genotypes reveal inter-strain incompatibility and the evolution of recombination. PLoS Genet. 7:e1002174. 10.1371/journal.pgen.1002174 PMC3136444 21779179

[evad055-B69] Sánchez-Ramírez S , Weiss JG, Thomas CG, Cutter AD. 2021. Widespread misregulation of inter-species hybrid transcriptomes due to sex-specific and sex-chromosome regulatory evolution. PLoS Genet. 17:e1009409. 10.1371/journal.pgen.1009409 PMC7968742 33667233

[evad055-B70] Satokangas I , Martin SH, Helanterä H, Saramäki J, Kulmuni J. 2020. Multi-locus interactions and the build-up of reproductive isolation. Philos Trans R Soc Lond B Biol Sci. 375:20190543. 10.1098/rstb.2019.0543 PMC7423273 32654649

[evad055-B71] Schiffman JS , Ralph PL. 2022. System drift and speciation. Evolution 76:236–251. 34529267 10.1111/evo.14356 PMC9292711

[evad055-B72] Shen SQ , Turro E, Corbo JC. 2014. Hybrid mice reveal parent-of-origin and *cis*- and *trans*-regulatory effects in the retina. PLoS One. 9:e109382. 10.1371/journal.pone.0109382 PMC4207689 25340786

[evad055-B73] Signor SA , Nuzhdin SV. 2018. The evolution of gene expression in *cis* and *trans*. Trends Genet. 34:532–544. 29680748 10.1016/j.tig.2018.03.007 PMC6094946

[evad055-B74] Stern DL . 2000. Evolutionary developmental biology and the problem of variation. Evolution 54:1079–1091. 11005278 10.1111/j.0014-3820.2000.tb00544.x

[evad055-B75] Strome S , Kelly WG, Ercan S, Lieb JD. 2014. Regulation of the X chromosomes in Caenorhabditis elegans. Cold Spring Harb Perspect Biol. 6(3):a018366. 10.1101/cshperspect.a018366 PMC3942922 24591522

[evad055-B76] Sundararajan V , Civetta A. 2011. Male sex interspecies divergence and down regulation of expression of spermatogenesis genes in Drosophila sterile hybrids. J Mol Evol. 72:80–89. 21079940 10.1007/s00239-010-9404-5

[evad055-B77] Takahasi KR , Matsuo T, Takano-Shimizu-Kouno T. 2011. Two types of cis - trans compensation in the evolution of transcriptional regulation. Proc Natl Acad Sci U S A. 108:15276–15281. 21876147 10.1073/pnas.1105814108 PMC3174679

[evad055-B78] Thomas CG , et al 2015. Full-genome evolutionary histories of selfing, splitting, and selection in *Caenorhabditis*. Genome Res. 25:667–678. 25783854 10.1101/gr.187237.114 PMC4417115

[evad055-B79] True JR , Haag ES. 2001. Developmental system drift and flexibility in evolutionary trajectories. Evol Dev. 3:109–119. 11341673 10.1046/j.1525-142x.2001.003002109.x

[evad055-B80] Tulchinsky AY , Johnson NA, Porter AH. 2014. Hybrid incompatibility despite pleiotropic constraint in a sequence-based bioenergetic model of transcription factor binding. Genetics 198:1645–1654. 25313130 10.1534/genetics.114.171397 PMC4256777

[evad055-B81] Turelli M , Moyle LC. 2007. Asymmetric postmating isolation: Darwin's corollary to Haldane's rule. Genetics 176:1059–1088. 17435235 10.1534/genetics.106.065979 PMC1894575

[evad055-B82] Verta J-P , Landry CR, MacKay J. 2016. Dissection of expression-quantitative trait locus and allele specificity using a haploid/diploid plant system–insights into compensatory evolution of transcriptional regulation within populations. New Phytol. 211:159–171. 26891783 10.1111/nph.13888

[evad055-B83] Wang Z , et al 2015. Evolution of gene regulation during transcription and translation. Genome Biol Evol. 7:1155–1167. 25877616 10.1093/gbe/evv059 PMC4419805

[evad055-B84] Wittkopp PJ , Haerum BK, Clark AG. 2004. Evolutionary changes in *cis* and *trans* gene regulation. Nature 430:85–88. 15229602 10.1038/nature02698

[evad055-B85] Wolf JBW , Lindell J, Backström N. 2010. Speciation genetics: current status and evolving approaches. Philos Trans R Soc Lond B Biol Sci. 365:1717–1733. 20439277 10.1098/rstb.2010.0023 PMC2871893

[evad055-B86] Woodruff GC , Eke O, Baird SE, Félix MA, Haag ES. 2010. Insights into species divergence and the evolution of hermaphroditism from fertile interspecies hybrids of Caenorhabditis nematodes. Genetics 186(3):997–1012. 20823339 10.1534/genetics.110.120550 PMC2975280

[evad055-B87] Wray GA . 2007. The evolutionary significance of *cis*-regulatory mutations. Nat Rev Genet. 8:206–216. 17304246 10.1038/nrg2063

[evad055-B88] Wu CI , Davis AW. 1993. Evolution of postmating reproductive isolation: the composite nature of Haldane's rule and its genetic bases. Am Nat. 142:187–212. 19425975 10.1086/285534

[evad055-B89] Xie D , et al 2022. Genetic exchange with an outcrossing sister species causes severe genome-wide dysregulation in a selfing Caenorhabditis nematode. Genome Res. 32(11-12):2015–2027. 36351773 10.1101/gr.277205.122 PMC9808620

[evad055-B90] Yin D , et al 2018. Rapid genome shrinkage in a self-fertile nematode reveals sperm competition proteins. Science 359:55–61. 29302007 10.1126/science.aao0827 PMC5789457

