# Supplementary material for: Regulatory Divergence as a Mechanism for X-Autosome Incompatibilities in Caenorhabditis Nematodes
Source: Genome Biol Evol. 2023 Apr 4;15(4):evad055. doi: 10.1093/gbe/evad055 (PMC10147328; doi:10.1093/gbe/evad055)
Supplement: evad055_Supplementary_Data [file evad055_supplementary_data.zip › Viswanath-Cutter_Supplementary-Figures-and-legends_27_2_23.docx]

**SUPPLEMENTARY MATERIALS**

**Regulatory divergence as a mechanism for X-autosome incompatibilities in *Caenorhabditis* nematodes**

Athmaja Viswanath, Asher D. Cutter ^*^

Department of Ecology and Evolutionary Biology

University of Toronto

25 Willcocks Street, Toronto, ON M5S 3B2, Canada

*Corresponding author: E-mail: [asher.cutter@utoronto.ca](mailto:asher.cutter@utoronto.ca)


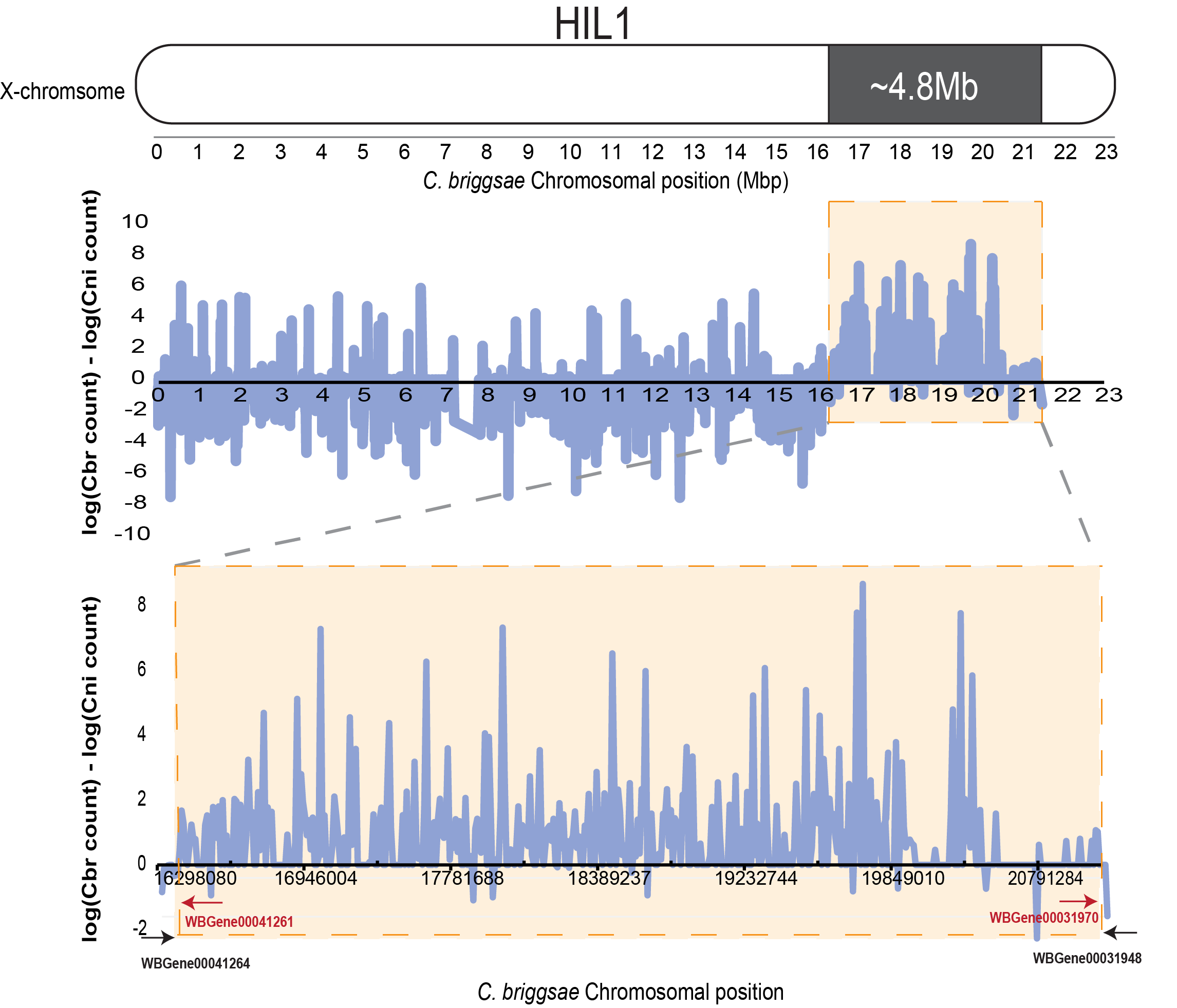


**Supplementary Figure S1: Confirmation of introgression boundary for HIL1 (strain ZZY10330).** Plot showing difference in log_2_(mean read count + 0.1) between readcounts obtained from mapping using *C. briggsae* reference genome and readcounts obtained from mapping using the *C. nigoni* reference genome for each gene along the X-chromosome (*C. briggsae* position). A positive value for this difference between readcounts indicates that the gene maps better to *C. briggsae* reference genome and hence is likely part of the C*. briggsae* X-chromosome i.e., part of introgressed region. A cluster of positive values around the expected introgression region (region within black box) was observed on the right arm of the X-chromosome. This region was used to define the introgression boundaries and confirmed the presence of ~4.8Mb fragment (*C. briggsae* positions 16.39 Mb to 21.26 Mb) defined by the genes WBGene00041261 at 16392964bp on the left and WBGene00031970 at 21269250bp on the right of the fragment introgressed in HIL1 from *C. briggsae*.
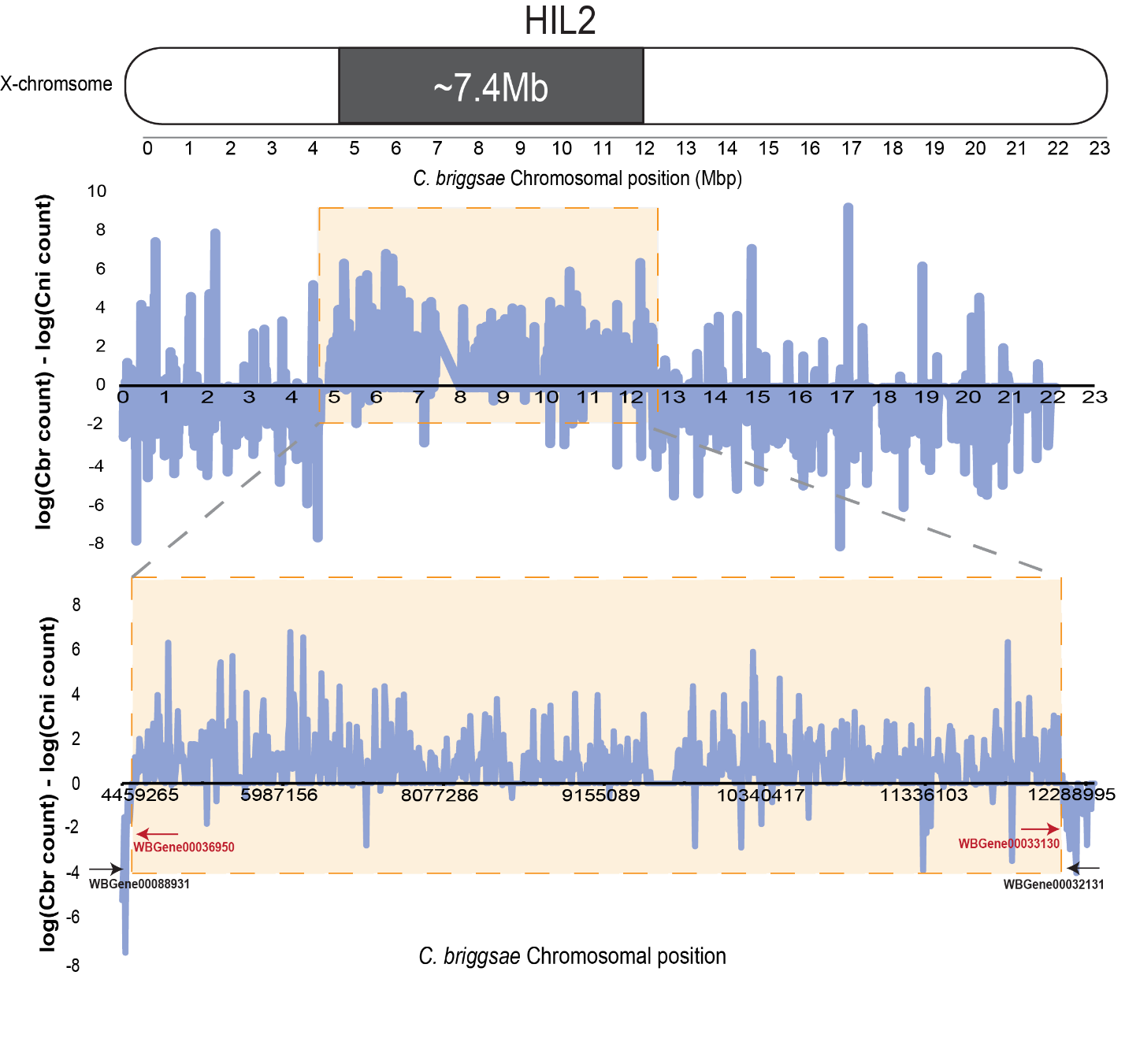


**Supplementary Figure S2: Mapping introgression boundary for HIL2 (strain ZZY10307).** A cluster of positive values was observed in the middle of the X-chromosome (region within black box). This region confirmed the presence of ~7.4Mb fragment (*C. briggsae* positions 4.74 Mb to 12.16 Mb) defined by the genes WBGene00036950 at 4742872bp on the left and WBGene00032130 at 12160549bp on the right of the *C. briggsae* fragment introgressed in HIL2.


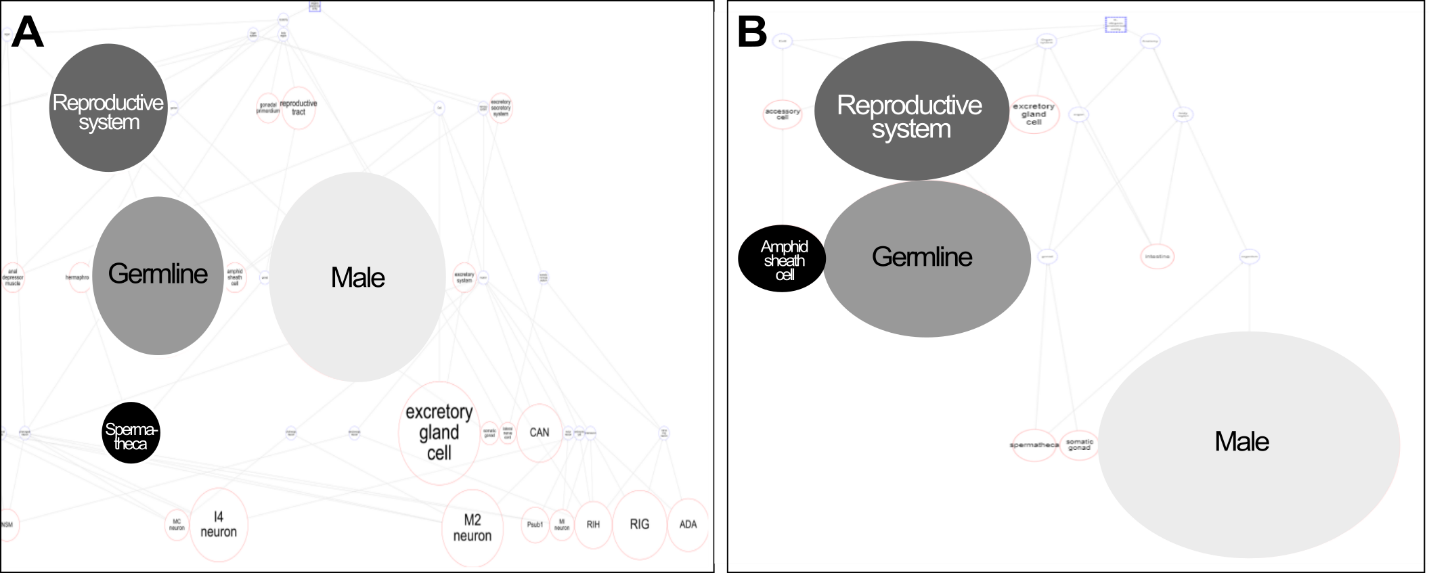


**Supplementary Figure S3: Tissue enrichment analysis (TEA) of downregulated genes in the shared genomic region**. The downregulated genes that have lower expression in HILs than in a pure *C. nigoni* genetic background were also enriched for male specific, reproductive and germline genes. Tissue enrichment analysis of downregulated genes in HIL1 (strain ZZY10330) (A) and HIL2 (strain ZZY10307) (B) reveal patterns that are consistent with previous analysis by (Li et al. 2016). TEA conducted using Wormbase enrichment analysis (n_HIL1_ = 1253 downregulated genes of 2229 DEGs among 10473 total genes; n_HIL2_ = 1549 downregulated genes of 2543 DEGs among 10541 genes) (Angeles-Albores et al. 2016, 2018).


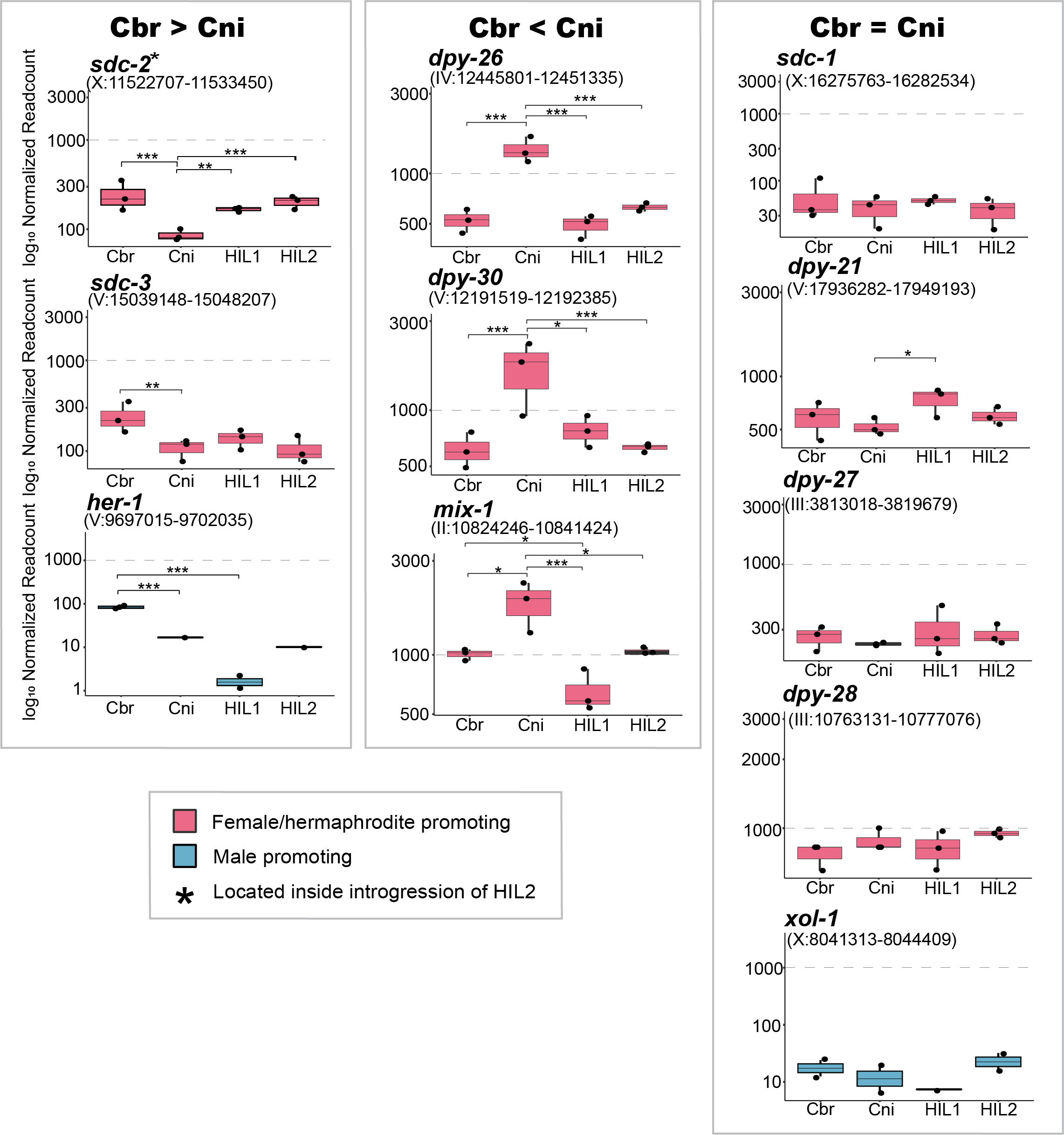


**Supplementary Figure S4: Expression of dosage comepnsation-related genes.** We analysed 11 genes related to dosage compensation in *C. elegans*. Nine of these 11 genes were female/hermaphrodite-promoting (*sdc-1, sdc-2, sdc-3*, *dpy-21*, *dpy-26, dpy-27, dpy-28, dpy-30*, and *mix-1*) and two were male-promoting (*xol-1*, *her-1*). We identified orthologous genes across *C. nigoni* and *C. briggsae* for these 11 genes using the Biomart tool of Wormbase Parasite (Howe et al. 2017), performed a differential gene expression analysis separately for these genes across parents and HILs, and categorised the genes based on the expression difference between *C. briggsae* and *C. nigoni* wildtype samples. Most genes showed equal expression across *C. briggsae* and *C. nigoni* (*sdc-1*, *dpy-21*, *dpy-27*, *dpy-28,* and *xol-1*); four of the 11 genes exhibited higher expression in *C. briggsae* (*sdc-2*, *sdc-3,* and *her-1*), while the rest showed higher expression in *C. nigoni* (*dpy-26*, *dpy-30,* and *mix-1*). However, we did not observe a clear pattern of expression for these genes, making it difficult to draw conclusions about the disruption of dosage compensation as an underlying mechanism of hybrid male sterility in this system.
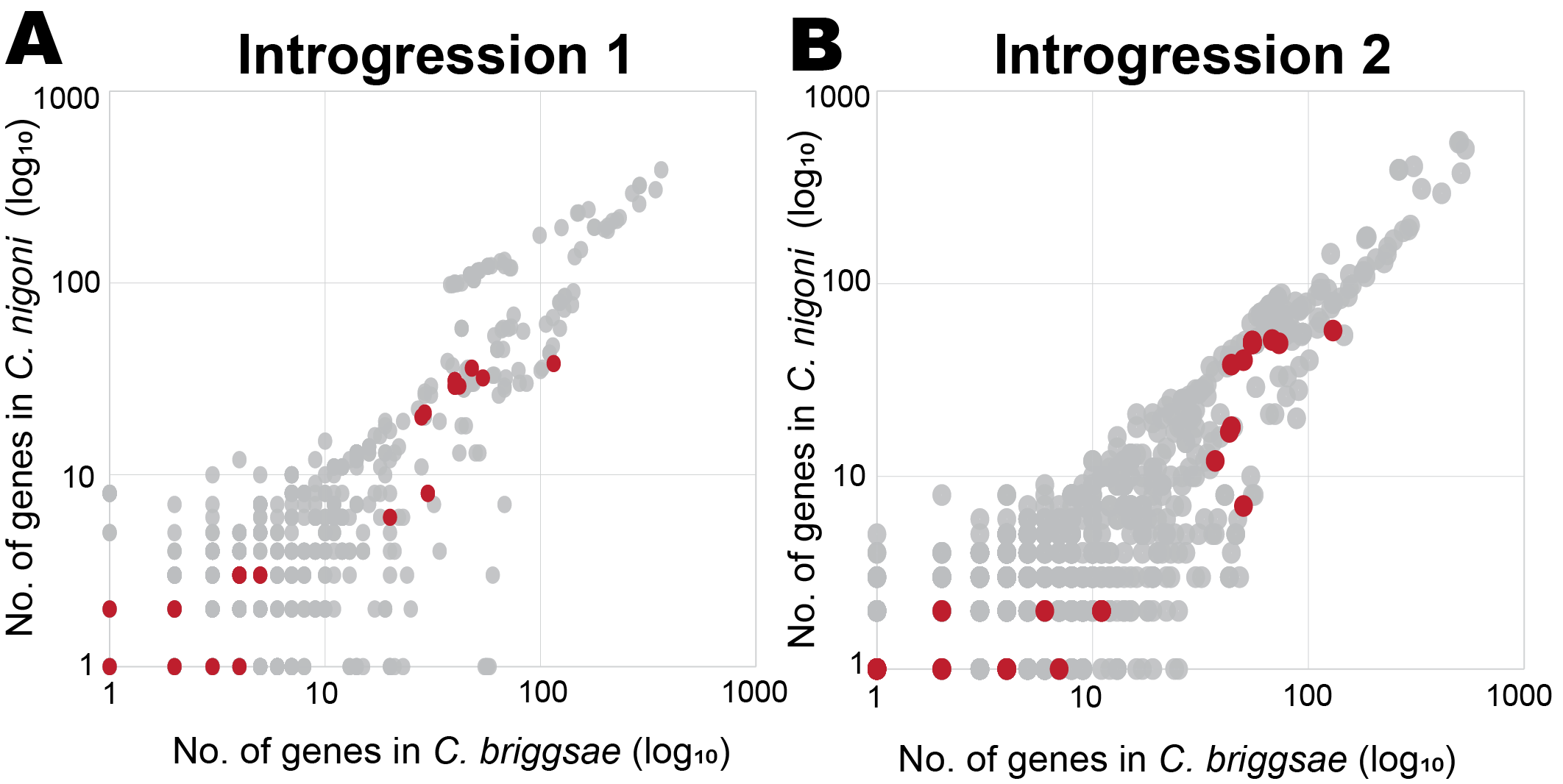


**Supplementary figure S5: Representation of gene ontology categories between syntenic regions of each X-linked introgression region.** Genes per gene ontology category for syntenic regions of *C. briggsae* and *C. nigoni* in X-linked introgression 1 (A; see HIL strain ZZY10330) and introgression 2 (B; see HIL2 strain ZZY10307). The red circles represent genes corresponding to categories related to RNA activity and transcription. The grey circles represent all other categories.

**Supplementary Tables**

All described tables are available in the file Viswanath-Cutter_Supplementary-Tables.xlsx.

**Supplementary Table legends**

**Table S1: Readcounts used to identify introgression boundaries of HIL1 strain ZZY10330**

Table contains readcounts for X-linked genes in all three replicates of *C. briggsae*, *C. nigoni,* and HIL1. For genes in HIL1, readcounts were obtained by mapping using both *C. briggsae* and *C. nigoni* reference genomes. The difference in mapped reads between them (log_2_(mean readcount + 0.1)) was used as a metric to identify introgression boundaries, with positive values indicating that the gene mapped better to *C. briggsae* than *C. nigoni* and hence is likely part of the introgressed region. The genes marking the boundary of the introgressed region are highlighted

**Table S2: Readcounts used to identify introgression boundaries of HIL2 strain ZZY10307**

Table contains readcounts for X-linked genes in all three replicates of *C. briggsae*, *C. nigoni,* and HIL2. For genes in HIL2, readcounts were obtained by mapping using both *C. briggsae* and *C. nigoni* reference genomes. The difference in mapped reads between them (log_2_(mean readcount + 0.1)) was used as a metric to identify introgression boundaries, with positive values indicating that the gene mapped better to *C. briggsae* than *C. nigoni* and hence is likely part of the introgressed region.

**Table S3: Autosomal and X-linked genes in the shared genomic region show similar patterns of overlap in inheritance across HILs**

Table shows the inheritance pattern across autosomal and X-linked genes present in the common genomic region across HILs. Overall, 10361 genes had detectable expression in both HILs in the shared genomic region (n_HIL1_ = 10361 of 10473; n_HIL2_ = 10361 of 10541) out of which 9598 genes were present on the autosomes and 763 genes were present on the X-chromosome. Overall, ~65% and ~72% of the autosomal and X-linked genes, respectively, were categorised in the same inheritance category across both HILs (n_autosomal overlap_ = 6274 of 9598; n_X-linked overlap_ = 551 of 763).

**Table S4: Genes in the shared genomic region show similar patterns of overlap in regulatory divergence profiles across HILs**

Table shows the regulatory divergence across HILs for genes present in the shared genomic region. Out of the 10361 genes showing expression in both HILs, 1276 genes exhibited the same regulatory divergence profiles across both HILs. Of these 1276 genes 828 and 448 genes were downregulated and upregulated respectively.

**Table S5: Genes in the shared genomic region and their regulatory divergence profiles across HILs**

Table shows the distribution of all the commonly expressed genes across both HILs. It shows the number of genes in each regulatory divergence category in each HIL including both genes overlapping and non-overlapping in regulatory divergence profiles across HILs.

**Table S6: Gene ontology terms for genes present in introgression 1**

Table depicting genes categorised into different gene ontology terms. Genes within the syntenic introgression region of HIL1 (*C. briggsae* X-chromosome positions 16,392,964 bp to 21,277,039 bp) and under the same GO terms are compared across *C. briggsae* and *C. nigoni*. The GO terms are broadly divided into "RNA-related” terms which includes genes related to RNA processing and transcription and “Other” which includes all other GO terms.

**Table S7: Gene ontology terms for genes present in introgression 2**

Table depicting genes categorised into different gene ontology terms. Genes within the syntenic introgression region of HIL2 (*C. briggsae* X-chromosome positions 4,742,872 bp to 12,165,712 bp) and under the same GO terms are compared across *C. briggsae* and *C. nigoni*. The GO terms are broadly divided into "RNA-related” terms which includes genes related to RNA processing and transcription and “Other” which includes all other GO terms.

**References**

Angeles-Albores D, Lee RY, Chan J, Sternberg PW. 2016. Tissue enrichment analysis for C. elegans genomics. BMC bioinformatics. 17:1–10.

Angeles-Albores D, Lee RYN, Chan J, Sternberg PW. 2018. Two new functions in the WormBase enrichment suite. microPublication Biology.

Howe KL, Bolt B J, Shafie M, Kersey P, Berriman M. 2017. WormBase ParaSite− a comprehensive resource for helminth genomics. Mol. Biochem. Parasitol, 215, 2-10.

Li R et al. 2016. Specific down-regulation of spermatogenesis genes targeted by 22G RNAs in hybrid sterile males associated with an X-Chromosome introgression. Genome Res. 26:1219–1232. doi: 10.1101/gr.204479.116.

Raudvere U, Kolberg L, Kuzmin I, Arak T, Adler P, Peterson H, Vilo J. 2019. g: Profiler: a web server for functional enrichment analysis and conversions of gene lists (2019 update). Nucleic Acids Res. 47(W1), W191-W198.
